# Emergence of carbapenem-resistant *Phytobacter diazotrophicus* in hospital effluent in Brazil

**DOI:** 10.3389/fmicb.2026.1859561

**Published:** 2026-07-03

**Authors:** Inayara de Sousa, Damaris Krul, Bianca Ribeiro da Silva Negoseki, Antônio Cavalcante de Souza Júnior, Isabelle Christine Rodrigues Marinho, Adriele Celine Siqueira, Jéssica Brustolim Lazarotto, Dany Mesa, Danieli Conte, Libera Maria Dalla-Costa

**Affiliations:** 1 Faculdades Pequeno Príncipe (FPP), Curitiba, Paraná, Brazil; 2 Instituto de Pesquisa Pelé Pequeno Príncipe (IPPPP), Curitiba, Paraná, Brazil; 3 Companhia de Saneamento do Paraná (Sanepar), Curitiba, Paraná, Brazil; 4 Hospital Pequeno Príncipe (HPP), Curitiba, Paraná, Brazil

**Keywords:** *bla*_KPC-2_, environmental microorganisms, hospital effluent, one health, *Phytobacter*

## Abstract

**Background:**

*Phytobacter* spp. have recently emerged as opportunistic pathogens in vulnerable patients and are increasingly recognized as carriers of clinically relevant carbapenemase genes, particularly *bla*_KPC-2_. *Phytobacter diazotrophicus* is the most frequently recovered species from clinical specimens in cases of infections. Despite this growing clinical importance, little is known about the presence, genomic characteristics, and environmental dissemination of *Phytobacter* spp. outside clinical settings. Hospital effluent is recognized as an important environmental pollutant and source of antimicrobial-resistant bacteria carrying resistance genes. However, the occurrence and genomic context of carbapenemase-producing *P. diazotrophicus* in hospital wastewater remain largely unexplored, particularly in Brazil, where *bla*_KPC-2_ is endemic. Therefore, this study aimed to isolate and characterize carbapenem-resistant *P. diazotrophicus* from the effluent of a pediatric hospital in Brazil.

**Methods:**

Wastewater samples were collected at Pequeno Príncipe Hospital, Curitiba Brazil. *Phytobacter diazotrophicus* isolates were isolated and identified using matrix-assisted laser desorption ionization time-of-flight mass spectrometry (MALDI-TOF MS). Whole-genome-based analysis was performed to confirm the identity of *P. diazotrophicus* isolates, and their antimicrobial susceptibility was tested using antimicrobial assays. Expression of the *bla*_KPC_ gene was confirmed via polymerase chain reaction.

**Results:**

MALDI-TOF MS revealed 345 microorganisms, and whole-genome comparisons supported the assignment of five strains to *P. diazotrophicus*. The resistance phenotypes determined via antimicrobial assays were consistent with the genomic antimicrobial resistance profiles, and two isolates (PHY1 and PHY5) exhibited resistance to carbapenems. Genomic analysis revealed the presence of *bla*_KPC-2_ in these two isolates.

**Conclusion:**

To the best of our knowledge, this study provides the first evidence of *bla*_KPC-2_ harboring *P. diazotrophicus* in hospital effluent in Brazil. The detection of carbapenemase-producing bacteria in hospital wastewater underscores the importance of effluent surveillance as a strategy to monitor and mitigate the environmental dissemination of antimicrobial resistance.

## 1 Introduction

Bacteria belonging to the genus *Phytobacter* inhabit a wide range of ecological niches, including clinical environments, in which they cause healthcare-associated infections. Owing to genetic similarity among the genera of the family *Enterobacteriaceae*, *Phytobacter* isolates are often misidentified as *Pantoea*, *Kluyvera, Enterobacter*, *Cronobacter*, *Citrobacter,* or *Kosakonia*.

Additionally, the taxonomic classification of *Phytobacter* has undergone considerable revisions. First described in 2008, *Phytobacter* and its type species *Phytobacter diazotrophicus* were initially identified as an endophytic nitrogen-fixing bacterium from wild rice in China (Zhang et al., 2008). Epidemiological studies conducted in Europe, Asia, and the Americas have consistently identified *P. diazotrophicus* as the most frequently recovered species from clinical specimens, particularly in cases of bloodstream-, respiratory tract-, and device-associated infections (Smits et al., 2022). Multiple outbreaks of *P. diazotrophicus* have been attributed to contaminated medical devices, parenteral nutrition solutions, and hospital water systems, thereby emphasizing their capacity for environmental persistence and nosocomial transmission. Furthermore, isolates associated with a multi-state sepsis outbreak in Brazil were classified as *P. diazotrophicus* and led to the description of a novel species (*Phytobacter ursingii*), which was identified among clinical strains from the United States (Pillonetto et al., 2018).

Like other members of the *Enterobacteriaceae* family, *Phytobacter* species can acquire and spread antimicrobial resistance (AMR) genes via mobile genetic elements (Kubota et al., 2023). This further contributes to the growing global AMR crisis, thereby posing threat to human, animal, and environmental health within the One Health framework.

Although antimicrobials have revolutionized modern medicine, their excessive and inappropriate use has accelerated the emergence and spread of resistant microorganisms. In the hospital settings, large quantities of these drugs are used daily, both for treating infections and for surgical prophylaxis. As a result, antimicrobial residues, as well as resistant bacteria and AMR genes, are excreted through patients’ feces and urine, thereby entering hospital effluents. From a One Health perspective, AMR also represents a considerable environmental problem, especially in aquatic ecosystems. In agriculture and aquaculture, antimicrobials are widely used for the treatment and prevention of animal diseases, which are subsequently being released into nearby soil and water bodies. Thus, resistant bacteria and genes are frequently detected in rivers, lakes, and oceans, with hospital effluents, agricultural runoff, and urban wastewater being the primary sources. Once in the environment, these contaminants spread through aquatic ecosystems, reaching animals and humans via contact with water or the food chain, thereby highlighting the interconnection between human, animal, and environmental health within the One Health framework. Furthermore, even advanced wastewater treatment systems that include primary sedimentation, aeration, and anaerobic treatment may not completely remove these contaminants, contributing to their persistence in the environment (Arnold et al., 2011).

The emergence and spread of *Enterobacteriaceae* that are resistant to third-generation cephalosporins or carbapenems pose a critical threat to public health. Owing to their clinical relevance and the limited therapeutic options available, the World Health Organization (WHO) has classified these organisms as high-priority pathogens for the research and development of new antimicrobial agents (Sati et al., 2025). This resistance is generally associated with the production of *β*-lactamases. Among these, *Klebsiella pneumoniae* carbapenemase (KPC) has attracted particular attention. Since its first report in the United States (Yigit et al., 2001), more than 280 KPC variants (KPC-1 to KPC-284) (http://bldb.eu/, last updated April 15, 2026), have been identified worldwide. In South America, KPC has become endemic in countries such as Argentina, Brazil, and Colombia, where new mutations in the KPC-2 enzyme are increasingly being reported (Lee et al., 2016).

The widespread dissemination of KPC variants is not limited to *K. pneumoniae*. Carbapenemase genes, such as *bla*_IMP-6_ (Smits et al., 2022)*, bla*_NDM-1_ (Kubota et al., 2023), and *bla*_KPC-2_ (Almuzara et al., 2024), have also been reported in *Phytobacter* species, thus highlighting the expanding diversity of environmental and opportunistic hosts. However, the occurrence and genomic context of carbapenemase-producing *P. diazotrophicus* in hospital wastewater remain largely unexplored, particularly in Brazil, where *bla*_KPC-2_ is endemic. Given the endemicity of *bla*_KPC-2_ in Brazil, we hypothesized that hospital wastewater could harbor carbapenem-resistant *P. diazotrophicus* carrying clinically important carbapenemase genes. Therefore, this study aimed to isolate and characterize carbapenem-resistant *P. diazotrophicus* isolates recovered from the effluent of a pediatric hospital in Curitiba, Brazil.

## 2 Materials and methods

### 2.1 Sample collection and processing

Wastewater samples were collected at Pequeno Príncipe Hospital (HPP), a 369-bed tertiary pediatric teaching hospital located in southern Brazil, from two discharge points of chlorinated effluent in September 2022 (spring), January (summer), June (autumn), and August (winter) of 2023. The hospital has an average water consumption of 6,200 m^3^ per month and generates approximately 3,350 m^3^ of wastewater per month (ranging from 100 to 200 m^3^ per day). The hospital effluent is treated at the Belém Wastewater Treatment Plant (WWTP), and the treated effluent is continuously discharged into the Iguaçu River at an average flow rate of 60–70 m^3^/min. HPP, the largest pediatric hospital in Brazil and internationally recognized as the first exclusively pediatric hospital in Latin America, was selected due to its high patient turnover, intensive antimicrobial use, and substantial wastewater generation, which may contribute to the selection and dissemination of antimicrobial-resistant bacteria, including *Phytobacter* spp.

For each season, two 1-L samples of treated effluent were collected in sterile glass bottles, transported on ice, and processed within 6 h using the membrane filtration method based on a previously described method (Krul et al., 2025). Distinctly colored colonies were selected from chromogenic urinary tract infection (UTI) agar supplemented with meropenem (1 mg/L) or ceftazidime (2 mg/L) and subcultured onto fresh antibiotic-supplemented UTI agar. Thereafter, isolated single colonies were transferred to trypticase soy agar and subsequently stored at −80 °C in brain-heart infusion broth containing 25% (v/v) glycerol for further screening of extended-spectrum *β*-lactamase (ESBL) and carbapenemase production.

### 2.2 Characterization of bacterial isolates and AMR genes

Microorganisms were isolated from the samples collected and identified using matrix-assisted laser desorption ionization time-of-flight mass spectrometry (MALDI-TOF MS) with a Microflex LT Biotyper 3.0 system (Bruker Daltonics, Bremen, Germany). Whole-genome sequence (WGS) comparisons were performed based on average nucleotide identity (ANI) to identify *P. diazotrophicus* strains.

An antimicrobial susceptibility test was performed using the disk diffusion method according to the guidelines and interpretive criteria of the European Committee on Antimicrobial Susceptibility Testing and the Brazilian Committee on Antimicrobial Susceptibility Testing (BrCAST) (BRCAST, 2025; EUCAST, 2025). The minimum inhibitory concentration (MIC) was determined using the broth microdilution method, in accordance with the recommendations of BrCAST and ISO 20776-1. Susceptibility to the novel antimicrobial combination of ceftazidime-avibactam was assessed using a gradient diffusion strip (Liofilchem, Teramo, Italy).

### 2.3 WGS and bioinformatics analysis

Genomic DNA was extracted from *P. diazotrophicus* isolates using a QIAamp DNA Mini Kit (Qiagen, Hilden, Germany) following the manufacturer’s instructions. DNA quantification was performed using both the NanoDrop One spectrophotometer (Thermo Fisher Scientific Inc., Waltham, MA, USA) and the Qubit fluorometer with the double-stranded DNA high-sensitivity assay kit (Thermo Fisher Scientific Inc).

WGS of paired-end libraries (2 × 300 bp) was performed using the Illumina NextSeq 1,000 platform (Illumina Inc., San Diego, CA, USA). Long-read WGS was performed using a MinION sequencer (Nanopore, Oxford, UK). MinION samples were prepared using the Rapid Barcoding Kit (SQKRBK114.24) and the Flow Cell Priming Kit (FLO-MIN114, R10) from Oxford Nanopore Technologies, following the manufacturer’s instructions.

Raw read quality was assessed using FastQC version 0.11.9, and quality-based trimming and filtering were performed using Trimmomatic version 0.39 (Bolger et al., 2014; Wingett and Andrews, 2018). Chromosomal DNA was *de novo* assembled using SPAdes version 3.14.1 (Bankevich et al., 2012) with the parameters --cov-cutoff 20 and --phred-offset 33. Assembly was performed using multiple k-mer values (21, 33, 55, 77, 99, and 127), which were incorporated into the SPAdes multi-k-mer iterative assembly strategy. Rather than selecting among independently generated assemblies, SPAdes integrate information from multiple k-mer sizes and applies graph-based optimization procedures to produce a final assembly with improved contiguity, reduced ambiguity, and reduced influence of sequencing errors and low-confidence regions. Assembly quality was evaluated based on standard assembly statistics, including total assembly length, sequencing coverage, number of contigs, and N50 values. Plasmid sequences were *de novo* assembled using Flye version 2.9.6 (Kolmogorov et al., 2019) with the --nano-hq option, and the --genome-size parameter was adjusted according to the estimated size of each plasmid to optimize assembly performance. For plasmid assemblies, circularization status was also assessed when applicable. The completeness and taxonomic classification of the resulting assemblies were assessed using DFAST (Tanizawa et al., 2016). Chromosomal and plasmid sequences were annotated using Prokka version 1.12 and Rapid Annotation using Subsystem Technology (RAST) version 1.073 (Aziz et al., 2008; Seemann, 2014). The resistome was predicted using Comprehensive Antibiotic Resistance Database version 3.2.7, ResFinder database version 4.1, PlasmidFinder version 2.0.1, and MobileElementFinder version 1.0.3 (Carattoli et al., 2014; Bortolaia et al., 2020; Johansson et al., 2021; Alcock et al., 2023). The complete genome assembly statistics are presented in Supplementary Table S1.

### 2.4 Plasmid profiling and transformation of *bla*_KPC_

Total plasmid DNA was extracted from *P. diazotrophicus* isolates harboring *bla*_KPC_ using the NucleoBond™Xtra Plus Midi Kit (Macherey-Nagel, Duren, Germany) following the manufacturer’s instructions. The extracted plasmids were introduced into *Escherichia coli* TOP10 cells (Thermo Fisher Scientific Inc.) by chemical transformation using 5X KCM buffer (0.5 M KCl, 150 mM CaCl_2_, and 250 mM MgCl_2_). Transformants were selected on Luria-Bertani agar supplemented with ampicillin (0.5 mg/L). Expression of the *bla*_KPC_ gene in transformants was confirmed via polymerase chain reaction (PCR) (Chen et al., 2022). For PCR-positive isolates, antimicrobial susceptibility was assessed using MIC testing as previously described (Poirel et al., 2011; Conte et al., 2022).

## 3 Results

A total of 345 microorganisms were isolated from the hospital wastewater samples and were identified using MALDI-TOF MS. Among these isolates, five were identified as *P. ursingii* and selected for further analysis. WGS comparisons based on ANI values of >97% supported the assignment of these five strains to *P. diazotrophicus*.

The resistance phenotypes of these strains determined via MIC testing were consistent with the genomic AMR profiles. Three isolates did not exhibit resistance to carbapenems, whereas one isolate showed resistance to polymyxins. Additionally, two isolates (PHY1 and PHY5) exhibited resistance to carbapenems. Furthermore, plasmid DNA from PHY1 and PHY5 strains was successfully transferred via chemical transformation into *E. coli* TOP10 cells. KPC-2 transformants exhibited cephalosporin resistance profiles similar to those of the donor strains, while remaining susceptible to carbapenems. These results are presented in Supplementary Table S2.

*P. diazotrophicus* isolates recovered from hospital effluent carried *bla*_CTX-M_ variants (CTXM-8 and CTX-M-15), *bla*_OXA_ (OXA-1 and OXA-9), and *bla*_TEM-1A_. Additionally, genes conferring resistance to aminoglycosides (*aadA2*) and fluoroquinolones (*qnrE1*, *qnrB1*, and *aac(6′)-Ib-cr5*) were identified, thereby supporting the observed multidrug resistance profiles (Supplementary Table S1).

**Figure 1:**
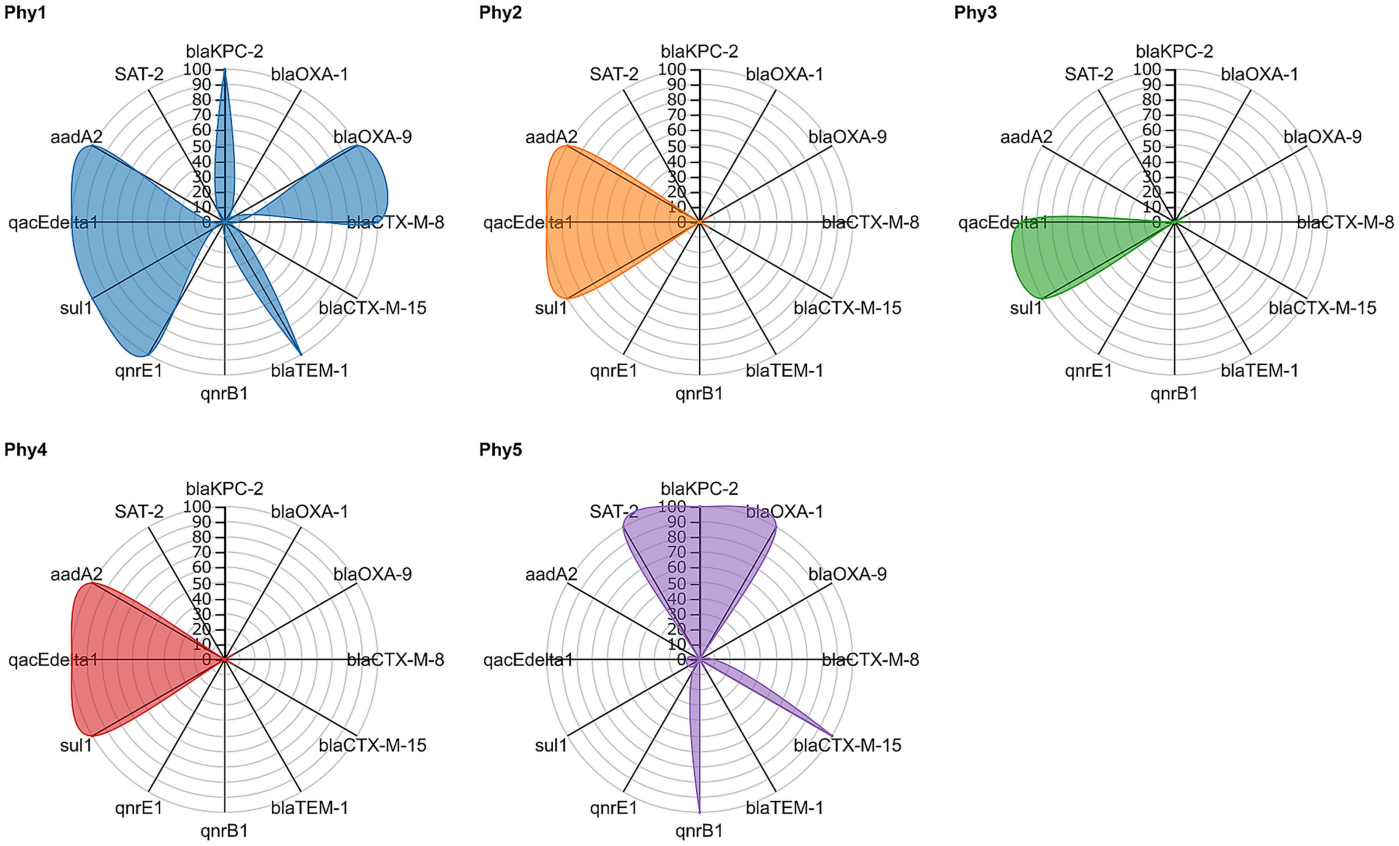
Distribution and prevalence of antimicrobial resistance genes across five distinct strains of *Phytobacter diazotrophicus* (PHY1–PHY5). Each radar chart depicts the resistance genes identified in a specific strain. Only genes with 100% sequence identity to reference sequences in the CARD database are shown, and different colors are used to distinguish among the five strains.

The main *β*-lactam resistance genes identified, *bla*_CTX-M-8_ (PHY1) and *bla*_CTX-M-15_ (PHY5), were located on IncM1 (p1PHY1) and IncHI2A (p2PHY5) plasmids, respectively (Supplementary Table S1 and Figure 1). All antimicrobial resistance genes reported in the Supplementary Table S1 and Figure 1 showed 100% sequence identity to their corresponding reference sequences in the CARD database.

The two identified isolates (PHY1 and PHY5) also harbored clinically significant plasmids carrying *bla*_KPC,_ located within transposon Tn*4401*, which is a structure that facilitates genetic mobilization. In both isolates, the presence of the KPC-2 enzyme was confirmed via WGS. BLASTn analysis revealed limited similarity between the IncU plasmid (p2PHY1) and a plasmid identified in *Raoultella ornithinolytica* from WWTP, showing 84% coverage and 99.9% nucleotide identity (GenBank accession No. AP022270.1).

**Figure 2:**
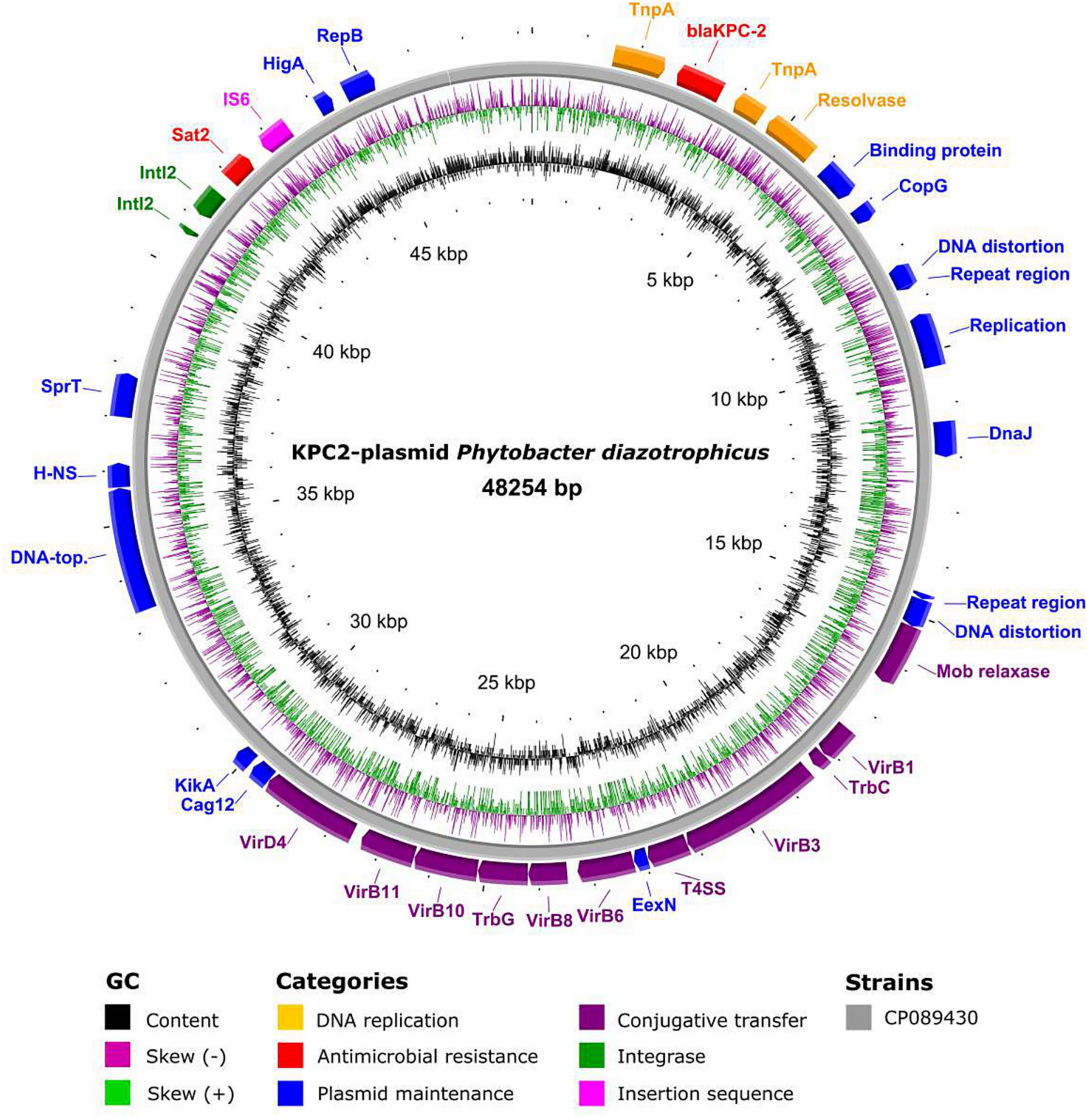
Circular map of the *bla*_KPC-2_-carrying plasmid recovered from the PHY5 strain isolated from the effluent of Pequeno Príncipe Hospital (HPP). The plasmid is compared with plasmid CP089430 owing to their high sequence similarity. Genes from PHY5 and CP089430 are shown as colored boxes according to their functional categories.

Notably, in the PHY5 isolate, *bla*_KPC-2_ was located on a conjugative IncX3–IncU hybrid plasmid (p1PHY5), which showed high sequence similarity (100% coverage and 99.99% identity) to a plasmid previously identified in 2011 in a clinical *Klebsiella pneumoniae* isolate from a hospital in São Paulo, Brazil (GenBank accession no. CP089430.1) (Figure 2).

## 4 Discussion

*Phytobacter* spp. have been increasingly recognized as opportunistic pathogens of clinical relevance (Smits et al., 2022), particularly following a 2013 outbreak in Brazil associated with contaminated parenteral nutrition. In this case, *P. diazotrophicus*, which was initially misidentified as *Pantoea* spp., was identified as the causative agent (Pillonetto et al., 2018). This case highlights the ability of *Phytobacter* to persist in environments traditionally considered sterile and the limitations of conventional identification methods.

Consistent with these identification challenges, the reclassification of our five *Phytobacter* isolates as *P. diazotrophicus* based on WGS highlights the limited discriminatory power of conventional proteomic approaches when resolving closely related taxa. Although MALDI-TOF is widely used owing to its speed and cost-effectiveness, its accuracy is dependent on the quality and comprehensiveness of reference databases (Clark et al., 2013). In contrast, sequencing-based approaches, particularly WGS, provide high taxonomic resolution, thereby reinforcing the importance of integrating genomic tools into diagnostic workflows for accurate species identification and surveillance (Gwinn et al., 2019). Accurate species identification is particularly important in this context, as it directly impacts the interpretation of AMR profiles and the epidemiological relevance of emerging pathogens.

The resistance phenotypes determined via MIC testing were consistent with the genomic AMR profiles identified in the isolates. In this context, two *Phytobacter* isolates (PHY1 and PHY5) were particularly noteworthy, as they harbored a diverse array of acquired resistance genes. These genes likely contribute to their cephalosporin and carbapenem resistance. Among the most relevant genes associated with these resistance phenotypes were *bla*_CTX-M_ and *bla*_KPC_.

CTX-M- type extended-spectrum *β*-lactamases are globally disseminated, with CTX-M-15 being the most prevalent variant (Bevan et al., 2017). These enzymes are reported in both clinical and environmental settings, and we previously detected them in *Enterobacterales* from hospital effluents, WWTP, and river water (Conte et al., 2017). Moreover, CTX-M variants have been described in *P. diazotrophicus*, including isolates associated with neonatal sepsis and those recovered from neonatal intensive care units (Milton et al., 2022; Hon et al., 2023; Huang et al., 2024).

In the current study, two variants (CTX-M-8 and CTX-M-15) were identified in two different *P. diazotrophicus* isolates from hospital effluent. These genes were located on IncM1 (p1PHY1) and IncHI2A (p2PHY5) plasmids. Notably, IncHI2A plasmids have previously been associated with clinically relevant resistance genes, including *mcr-9* and *bla*_IMP-4_ in *Phytobacter* spp. (Tumeo et al., 2025). Collectively, these findings suggest that *P. diazotrophicus* may contribute to the environmental dissemination of AMR genes, although extended conclusions are limited by the number of isolates analyzed.

Since the late 2000s, *bla*_KPC-2_ has emerged as the predominant determinant of carbapenem resistance in Brazil (Gonçalves et al., 2024) and has been widely disseminated among *Enterobacterales*, particularly *K. pneumoniae*, in both clinical and environmental settings, including hospital effluents (Krul et al., 2025). The detection of *bla*_KPC-2_ in *P. diazotrophicus* has only recently been reported, with the first description in 2024 involving a clinical isolate recovered from Argentina and confirmed via WGS (Almuzara et al., 2024). In the current study, *bla*_KPC-2_ was identified in two isolates. In PHY5, the gene was located on a conjugative plasmid previously described in a different genus that originated from a hospital-associated environment (Krul et al., 2025). This observation suggests the potential for horizontal gene transfer of clinically relevant resistance determinants across bacterial species and ecological niches.

In addition to previous reports of isolates harboring *bla*_NDM-1_ on IncA/C₂ plasmids (Kubota et al., 2023), our findings indicate that *P. diazotrophicus* isolates can also carry *bla*_KPC-2_-encoding plasmids. This further reinforces the potential of the genus as a reservoir and vector of clinically relevant AMR genes.

Hospital effluents are recognized as important hotspots for the selection and dissemination of AMR due to their high chemical and microbiological complexity, and the presence of antimicrobial residues resulting from intensive antibiotic use (Conte et al., 2017). In this context, mobile genetic elements play a critical role in the horizontal transfer of resistance determinants, including *bla*_KPC_, between clinically relevant and environmental bacteria, thereby strengthening the connection between clinical and environmental resistomes. While the present findings originate from a pediatric tertiary-care hospital, comparable selective pressures and AMR dissemination risks may also occur in other tertiary-care hospital environments, suggesting that the implications of these findings may extend beyond the specific setting investigated here.

The environmental dissemination of resistant microorganisms is further aggravated by the limited capacity of conventional WWTPs to efficiently remove antimicrobial-resistant bacteria, AMR genes, and pharmaceutical residues. At Hospital Pequeno Príncipe, the high number of hospitalizations and surgical procedures generates substantial antimicrobial selective pressure within the hospital effluent. The most frequently used antimicrobials in the institution include ceftriaxone, azithromycin, sulfamethoxazole-trimethoprim, meropenem, and cefepime, which may contribute to the selection and persistence of resistant microorganisms in wastewater.

Moreover, *Phytobacter* spp. are frequently associated with immunocompromised patients and individuals receiving parenteral nutrition, conditions commonly observed in pediatric tertiary-care hospitals. This scenario may favor the emergence and dissemination of opportunistic pathogens carrying clinically relevant resistance determinants.

Consequently, the continuous discharge of treated effluents into aquatic environments may facilitate the persistence and environmental circulation of AMR, representing a potential risk to ecosystem integrity and public health through exposure of animals and humans, as well as the possible contamination of water resources used for public supply.

## 5 Conclusion

From a One Health perspective, our findings suggest that *P. diazotrophicus* may represent a potentially under-recognized opportunistic pathogen and a possible environmental reservoir of clinically relevant AMR genes. The detection of multidrug-resistant isolates in hospital effluents, including those carrying ESBLs and carbapenemases, emphasizes the potential role of healthcare wastewater as an environment contributing to the dissemination of resistance. In Brazil, the lack of specific regulations for hospital effluent management, together with the incomplete removal of resistance determinants during wastewater treatment, may facilitate their environmental spread. These observations highlight the importance of further investigation, including studies addressing the occurrence and dissemination of these resistance determinants in surrounding environmental settings such as wastewater and water treatment systems receiving treated hospital effluent, as well as the need for integrated genomic surveillance, improved wastewater management policies, and coordinated One Health approaches.

## Data Availability

The datasets presented in this study can be found in online repositories. The names of the repository/repositories and accession number(s) can be found in the article/Supplementary material.
