# Supplementary material for: Emergence of carbapenem-resistant Phytobacter diazotrophicus in hospital effluent in Brazil
Source: Front Microbiol. 2026 Jul 3;17:1859561. doi: 10.3389/fmicb.2026.1859561 (PMC13376303; doi:10.3389/fmicb.2026.1859561)
Supplement: Supplementary file 1 [file Table_1.DOCX]

**Table S1**: Genomic characteristics and resistance genes of *P. diazotrophicus* isolated from hospital effluent.

| **Strain** | **MALDI-TOF ID** | **WGS ID** | **ANI (%)** | **Genome** | **Lenght (pb)** | **GC%** | **RNAs (n)** | **CDS** | **Inc group** | **Resistance genes** | **GenBank accession** |
| --- | --- | --- | --- | --- | --- | --- | --- | --- | --- | --- | --- |
| PHY1 | *P. ursingii* | *P. diazotrophicus* |  | chromossome | 5,861,005 | 52.9 | 89 | 5984 | - | *sul1, qacEdelta, aadA2* |  |
|  |  |  | 98.8 | Plasmid p1PHY1 | 124,78 | 44.8 | 0 | 158 | IncM1 | *bla*_TEM-1A_, *aadA1, aac(6')-Ib, qnrE1, bla*_OXA-9_, *bla*_CTX-M-8_ | [SAMN54910896](https://dataview.ncbi.nlm.nih.gov/object/63020438) |
|  |  |  | 97.83 | Plasmid p2PHY1 | 273,069 | 52.3 | 2 | 446 | IncU | *bla*_KPC-2_ |  |
| PHY2 | *P. ursingii* | *P. diazotrophicus* | 97.65 | chromossome | 5,447,437 | 53.0 | 83 | 5411 | - | *aadA2, qacEdelta1, sul1* | [SAMN54910897](https://dataview.ncbi.nlm.nih.gov/object/63020439) |
| PHY3 | *P. ursingii* | *P. diazotrophicus* | 97.62 | chromossome | 5,502,482 | 53.1 | 83 | 5491 | - | *qacEdelta1, sul1* | [SAMN54910898](https://dataview.ncbi.nlm.nih.gov/object/63020440) |
| PHY4 | *P. ursingii* | *P. diazotrophicus* | 97.66 | chromossome | 5,528,546 | 53.1 | 84 | 5509 | - | *aadA, qacEdelta1, sul1* | [SAMN54910899](https://dataview.ncbi.nlm.nih.gov/object/63020441) |
| PHY5 | *P. ursingii* | *P. diazotrophicus* |  | chromossome | 5,486,162 | 53.2 | 82 | 5459 | - | *sul1, qacEdelta1* |  |
|  |  |  | 98.65 | Plasmid p1PHY5 | 48,254 | 46.5 | 0 | 76 | IncX3 - IncU | *bla*_KPC-2_ |  |
|  |  |  | 97.65 | Plasmid p2PHY5 | 124,78 | 44.8 | 0 | 158 | IncHI2A | *bla*_CTX-M-15_*, aac(6')-Ib-cr5, bla*_OXA-1_*, catB3, aac(3)-IIa, qnrB1* | [SAMN54910900](https://dataview.ncbi.nlm.nih.gov/object/63020442) |
|  |  |  | 97.62 | Plasmid p3PHY5 | 43,437 | 53.4 | 0 | 71 | IncFIB(K) | *qnrB1* |  |
