# Supplementary material for: Emergence of carbapenem-resistant Phytobacter diazotrophicus in hospital effluent in Brazil
Source: Front Microbiol. 2026 Jul 3;17:1859561. doi: 10.3389/fmicb.2026.1859561 (PMC13376303; doi:10.3389/fmicb.2026.1859561)
Supplement: Supplementary file 2 [file Table_2.docx]

**Table S2**: Susceptibility profile of *P. diazotrophicus*, *E. coli* TOP10 and transformants.

| **Strains** | **MIC (µg/mL)** | | | | | | | | | | |
| --- | --- | --- | --- | --- | --- | --- | --- | --- | --- | --- | --- |
|  | **CAZ** | **CPM** | **IMI** | **MER** | **GEN** | **AMI** | **CST** | **POL** | **CIP** | **CAZ-AVI** |  |
| PHY1 | R | R | R | I | R | S | S | S | R | S |  |
| *E.coli* transformant PHY1, KPC-2 | R | S | S | S | S | S | S | S | I | S |  |
| PHY2 | S | S | S | S | S | S | S | S | S | S |  |
| PHY3 | S | S | S | S | S | S | R | S | S | S |  |
| PHY4 | S | S | S | S | S | S | S | S | S | S |  |
| PHY5 | R | R | S | R | R | S | S | S | R | S |  |
| *E.coli* transformant PHY5, KPC-2 | R | R | S | S | S | S | S | S | S | S |  |
| *E. coli* TOP10 | I | S | S | S | S | S | S | S | S | S |  |

CAZ, ceftazidime; CPM, cefepime; IMI, imipenem; MER, Meropenem; GEN, gentamicina; AMI, amicacin; CST, colistin; POL, polymyxin; CIP, ciprofloxacin; CAZ-AVI, ceftazidime-avibactam.
